# Pretherapeutic Serum Albumin as an Outcome Prognosticator in Head and Neck Adenoid-Cystic Carcinoma

**DOI:** 10.3390/biomedicines10010191

**Published:** 2022-01-17

**Authors:** Marlene Friedl, Stefan Stoiber, Faris F. Brkic, Lorenz Kadletz-Wanke

**Affiliations:** 1 Department of Otorhinolaryngology, Head and Neck Surgery, Medical University of Vienna, 1090 Vienna, Austria; marlene.friedl@hotmail.com; 2 Department of Pathology, Medical University of Vienna, 1090 Vienna, Austria; stefan.stoiber@meduniwien.ac.at; 3 Christian Doppler Laboratory for Applied Metabolomics, Medical University of Vienna, 1090 Vienna, Austria

**Keywords:** adenoid-cystic carcinoma, albumin, prognostic marker, survival

## Abstract

Background: A head and neck adenoid-cystic carcinoma is a rare malignant tumor arising from the salivary gland tissues. The long-term survival outcome is poor due to a high risk of recurrences and distant metastasis. The identification of prognostic markers could contribute to a better risk assessment of each patient. The aim of this study is to assess the potential prognostic value of serum albumin in patients with head and neck adenoid-cystic carcinomas. Patients and Methods: This retrospective cohort study included all patients treated for a head and neck adenoid-cystic carcinoma between 1993 and 1 June 2019 with available pretherapeutic albumin values and clinical follow-up data. The cohort was stratified into a high and low group according to the median albumin value. The log-rank test was used for comparing overall and disease-free survival. Results: A total of 37 patients with complete follow-up data and available pretreatment albumin values were available. The overall mortality and recurrence rates were 21.6% (*n* = 8) and 45.9% (*n* = 17), respectively. Survival was shorter in the low albumin group. In particular, the mean overall survival for the low and high albumin groups were 121.0 months and 142.8 months, respectively. However, the difference was not statistically significant (*p* = 0.155). A statistically significant difference was observed in context with disease-free survival (45.2 months, 95% confidence interval 31.7–58.8 months vs. 114.8 months, 95% confidence interval 79.3–150.4 months; *p* = 0.029). Conclusion: Our study suggests a potential prognostic value of serum albumin in patients with a head and neck ACC. A further, external validation of our results is warranted.

## 1. Introduction

An adenoid-cystic carcinoma (ACC) is a rare malignant tumor of the major and minor salivary glands. It is characterized by a slowly growing local disease but a high risk of recurrence and distant metastases [1]. The initial overall survival (OS) rates are acceptable. Manneli et al. reported estimated 5- and 10-year survival rates of 74% and 50%, respectively. However, local failure and distant metastases impose a major problem. In particular, the rates of local recurrence can range up to 85% [2]. Moreover, the reported rates of distant metastases are described as being up to 70% [3,4].

Patients with head and neck malignant tumors are often confronted with dysphagia due to dysfunction or obstruction in the oral cavity or upper digestive tract. This, combined with a loss of appetite due to chemotherapy or circulating tumor-associated factors, often leads to malnourishment or cachexia. The nutritional status can be further deteriorated by the side effects of therapy such as fatigue and nausea. A significant correlation between nutritional deficiencies and a worse survival outcome in cancer patients is known and has already been reported by several authors [5,6].

Overall, serum albumin is regarded as an important nutritional status marker [7]. Further factors may also influence the serum albumin level. Systemic inflammation as well as factors produced by malignant tumors can inhibit hepatic albumin production [7,8]. Moreover, the cancer-related inflammatory response leads to a loss in body cell mass and weight loss. These factors subsequently result in low serum albumin levels [6]. Additionally, an increased vasopermeability in cancer patients results in a higher albumin escape into the extracellular compartment [8].

The prognostic value of serum albumin has already been shown for different cancer entities. As reported in the review of Gupta et al., high serum albumin was a predictor for better survival in different cancer entities such as gastrointestinal tract cancer or lung cancer [8]. The prognostic value of albumin was assessed in patients with head and neck malignancies as well. In particular, low pretreatment serum albumin was linked to worse disease-free survival (DFS), cancer-specific survival and OS in head and neck squamous cell carcinomas [6]. However, the study cohort did not include patients with an ACC.

An easily available prognosticator could facilitate the counseling of ACC patients. However, this task is seemingly hindered by the fact that an ACC is a rare malignant tumor [1], which hampers the building of a large cohort and potentially results in missing several statistical differences.

To the best of our knowledge to date, no studies have analyzed the association between pretherapeutic serum albumin levels and survival in head and neck ACCs. Furthermore, efforts have been given to assess the prognostic capacity of other biomarkers. Liu et al. [9] analyzed the association of the myeloblastosis gene fusion as well as protein expression with outcomes in ACCs. However, they were not able to detect any significant prognostic relevance of the proposed biomarker. On the other hand, Bazarsad et al. [10] were able to present a significant association of the survival outcome with the expression of mutated ataxia telangiectasia and p53. Similarly, in a previous study by our group [11], a significant correlation of AF1q overexpression with an impaired survival in ACCs was shown. However, all these markers warrant a thorough immunohistological analysis. The literature still remains sparse regarding easily obtainable inflammatory or nutritional outcome biomarkers for patients with ACCs.

As all patients undergo a routine blood test before the start of therapy, the obtaining of serum albumin values seems straightforward. Therefore, in the current study, we aimed to investigate the association between pretreatment serum albumin levels and the survival outcome in patients with head and neck ACCs treated at our center. This could potentially contribute to sorting out patients with a poor outcome prior to the start of therapy.

## 2. Materials and Methods

This retrospective study included all patients with histologically verified ACCs and primarily treated ACCs at the Department of Otorhinolaryngology and Head and Neck Surgery at the Medical University of Vienna, Austria. The data were acquired using the medical charts of patients. All patients treated for ACCs in the period from 1993 to 2019 were included in the study. The date of the initial diagnosis, the localization, the TNM staging (AJCC 8th Edition, 2017), the therapy approach, OS and DFS were assessed. To assess the pretherapeutic values of albumin, routinely performed pretreatment laboratory tests were used (0–7 days prior to the start of therapy).

### 2.1. Statistics

A statistical analysis as well as the visualization of the results was performed with the Statistical Package for the Social Sciences (SPSS, IBM Corp. Released 2016. IBM SPSS Statistics for Windows, Version 24.0. IBM Corp., Armonk, NY, USA) The values of serum albumin were dichotomized into high and low according to the median value (>median was considered high). We considered the results to be statistically significant at *p* < 0.05, two-sided. Based on the histograms, a normal distribution of the data could be assumed. Therefore, descriptive data using mean and standard deviations (SD) were presented. The log-rank test was used to compare the survival times between the groups and these were presented using the mean and 95% confidence intervals (CI). We visualized the survival data using Kaplan–Meier curves. Due to a low number of events, a multivariable analysis was not performed.

### 2.2. Ethical Statement

The current study was performed according to the Helsinki Declaration and our institutional review ethics board approved the patient data sampling and analysis (Approval number 1262/2019).

## 3. Results

### 3.1. Patients

Thirty-seven patients with histologically verified ACCs and available pretherapeutic albumin values and complete follow-up data were included in the study. The mean age of the cohort was 57.2 years +/−14.2 years. Sixteen were male (43.3%) and twenty-one patients were female (56.7%).

An advanced local disease was observed in the majority of patients. A total of 22 (59.5%) had T4 and T3 tumors and a T2 or T1 tumor was diagnosed in 15 patients (40.5%). Six of the 37 patients (16.2%) had lymph node metastases (N+) and one patient (2.7%) suffered from distant metastases (M1) at the time of diagnosis. The majority of 28 out of the 37 patients received primary surgery (75.7%). Additional to primary surgery, 14 patients were treated with postoperative radiotherapy (37.8%) and one with postoperative chemoradiotherapy (2.7%). Radiotherapy was chosen as a primary therapy in 8 out of 37 patients (21.6%). Two of those eight patients received a concomitant chemotherapy. Systemic palliative chemotherapy was the only possible option for one patient with primary distant metastases (2.7%). Furthermore, the majority of tumors were located in the minor salivary glands of the head and neck area (67.6%, *n* = 25). Others were located in the sublingual (2.7%, *n* = 1), submandibular (16.2%, *n* = 6) and the parotid glands (13.5%, *n* = 5). The patient details, tumor characteristics and therapy approaches are shown in Table 1.

A value for the pretherapeutic serum albumin was available for all 37 noted patients. The mean pretreatment serum albumin value for the whole cohort was 43.5 g/L +/−4.1 g/L. As noted, we used the median value of the serum albumin for the cohort stratification. In particular, the median albumin value was 43.4 g/L. The low albumin group consisted of 19 patients (≤median) and the high group contained 18 patients (>median).

### 3.2. Survival

The mean OS and DFS were 58.1 months +/−43.5 months and 45.2 months +/−37.9 months, respectively. The overall mortality rate was 21.6% (*n* = 8) and the recurrence rate was 45.9% (*n* = 17). Furthermore, in 21.6% (*n* = 8) of patients, multiple recurrences were diagnosed during the follow-up.

The survival was compared between the two noted groups. As expected, the log-rank test revealed a shorter OS and DFS in the group with low pretreatment serum albumin. This difference was not significant for OS (mean OS 121.0 months, 95% CI 86.7–155.3 months vs. 142.8 months, 95% CI 116.4–169.1 months; *p* = 0.155). However, a statistically significant difference was calculated for DFS (mean DFS 45.2 months, 95% CI 31.7–58.8 months vs. 114.8 months, 95% CI 79.3–150.4 months; *p* = 0.029)

**Figure 1:**
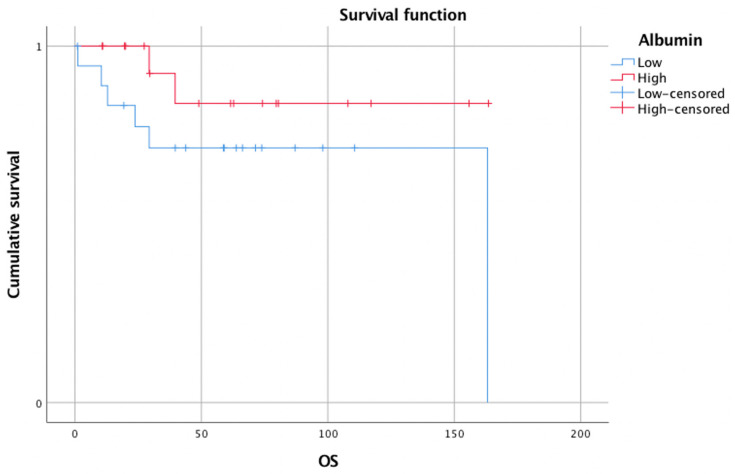
Kaplan–Meier survival curve for OS for patients stratified according to median pretherapeutic serum albumin value. The OS for the low albumin group (*n* = 19) was shorter than for the high albumin group (*n* = 18). A log-rank test revealed no significant difference (*p* = 0.155). OS: overall survival.

**Figure 2:**
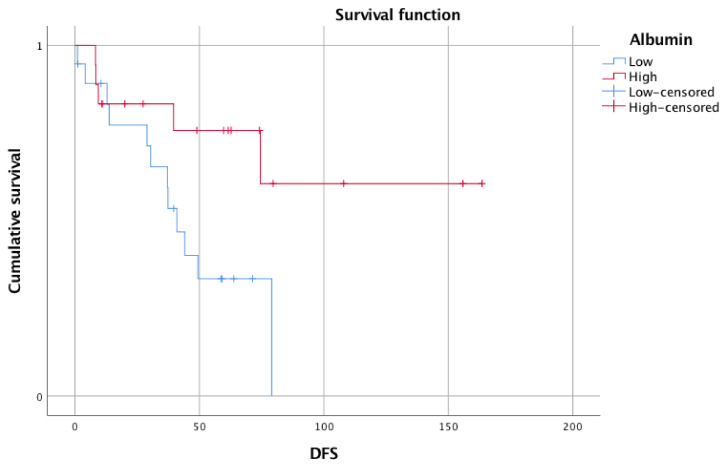
Kaplan–Meier survival curve for DFS for patients stratified according to median pretherapeutic serum albumin value. The DFS for the low albumin group (*n* = 19) was shorter than for the high albumin group (*n* = 18). A log-rank test revealed a statistically significant difference (*p* = 0.029). DFS: disease-free survival.

The visualization of the survival comparisons was performed with Kaplan–Meier survival curves and these are shown in Figure 1 for OS and Figure 2 for DFS.

## 4. Discussion

In this retrospective study, the authors aimed to assess the potential outcome prognostic value of pretreatment serum albumin in patients with head and neck ACCs. The first evidence of its potential prognostic relevance was delivered. In accordance with other studies analyzing pretreatment albumin as an outcome prognosticator in cancer patients [8,12,13,14], low serum albumin was associated with a worse survival in our cohort. In particular, a significantly shorter DFS was observed in patients with low serum albumin values. The prediction of DFS is of particular importance in ACC patients because local recurrences and distant spread are usually more of an issue than mortality in this patient group [1,2,3,4].

Malnutrition and subsequent cachexia are major problems for cancer patients, particularly for those in advanced stages. There are several factors contributing to malnutrition in patients with malignant tumors. In the case of head and neck malignancies, dysphagia is often observed and is mostly caused by obstruction of the upper digestive system or therapy-related sequalae. This subsequently leads to a reduced caloric intake. Moreover, cancer patients are in general confronted with inadequate intestinal absorption [15]. Importantly, weight loss can also occur as a side effect of chemotherapy, radiotherapy or as a result of cancer-related inflammation [15]. The association of cachexia and malnutrition with a poor survival outcome in cancer patients is well-known [16].

Albumin is a serum hepatic protein and is considered to reflect the nutritional status of the patient [17,18]. Hypoalbuminemia is a marker for cachexia or malnutrition in cancer patients. Other factors can contribute to hypoalbuminemia such as systemic inflammation and an inadequate protein intake. A reduced calorie and protein intake results in a lower albumin synthesis. Systemic inflammation leads to an increased vascular permeability and to a subsequent transcapillary escape of albumin [19,20]. Furthermore, tissue damage and necrosis could eventually lead to a decreased synthesis of albumin in the liver [21]. Other different reasons for cancer-related hypoalbuminemia have also been discussed. It was established by Babson and collaborators in 1954 that a tumor may trap the plasma proteins and utilize their degradation products for its own growth [22]. In summary, besides being an indicator for malnutrition, hypoalbuminemia also associates with cancer-associated inflammation as well as cancer progress. The most presumable reasons for hypoalbuminemia in patients with head and neck cancer or ACCs are, therefore, either malnutrition due to dysphagia, obstructions of the upper digestive system or cancer progress. Therefore, these could be hypothesized for our cohort as well.

Several studies have identified assessed serum albumin as a possible survival predictor in patients with malignant tumors, as shown in the review by Gupta and collaborators [8]. The meta-analysis of Asher and colleagues summarizes these results for patients with ovarian epithelial cancer. They showed that preoperative serum albumin independently predicted OS in these patients. In particular, they showed that for every 10 g/L loss of serum albumin, the summarized hazard ratio was 0.56 [23]. Importantly, Don et al. reported that the intravenous administration of albumin in critically ill patients with hypoalbuminemia did not improve their survival. Therefore, the cause of low albumin (e.g., cachexia) rather than the hypoalbuminemia itself could be seen as the cause for the poor outcome [19].

An association between the pretherapeutic albumin value and the survival outcome has also been reported for head and neck malignancies. In particular, Danan et al. were able to show that low serum albumin levels independently predicted a worse OS in patients with head and neck cancer [24]. This study included squamous cell carcinomas and melanomas. However, no cases of ACCs were described in the study. Similarly, Lim et al. analyzed the prognostic value of pretherapeutic serum albumin in 388 patients with head and neck squamous cell carcinomas. Patients with low pretreatment albumin levels had a six-fold higher risk of a worse survival outcome. However, as in the previously noted study, no patients with an ACC were included [6].

The prognostic relevance of other biomarkers for head and neck cancer was shown as well. Rassouli et al. analyzed several inflammatory and hematologic markers as potential outcome prognosticators in head and neck squamous cell carcinomas. They revealed that the neutrophil-to-lymphocyte ratio (NLR) independently predicted recurrence and that the platelet-to-lymphocyte ratio (PLR) was an independent prognostic marker for mortality [25].

In an earlier study by our group, we were able to show an association between the pretreatment NLR and the rate of recurrence in head and neck ACCs. A higher NLR was linked to the occurrence of multiple recurrences [26]. No further studies analyzing potential hematological or inflammatory markers as outcome prognosticators in patients with head and neck ACCs could be identified. In particular, the potential prognostic value of serum albumin has not yet been assessed for ACCs. Moreover, studies on the prognostic potential of other markers associated with malnutrition and systemic inflammation and/or malnutrition in ACCs are warranted; for example, absolute neutrophil or lymphocyte numbers or the NLR.

Our study had several limitations including the small number of patients included in the cohort. The main reason was the fact that an ACC is a low incidence malignancy [1]. Nevertheless, the number of included patients was comparable with other cohort studies on head and neck ACCs, which often face this kind of limitation [2,4,26,27]. Based on the relatively low number of included patients (and events in the survival analysis), a subgroup analysis—e.g., a Cox regression including confounders (e.g., therapy regimen or tumor staging)—could not be performed. Another important limitation was the retrospective study design. Hence, a possible confusion bias or a selection bias could not be excluded. Finally, we used the median albumin value as a cut-off for patient stratification. The validation of this value needs an external validation cohort.

## 5. Conclusions

We provided insights into the possible prognostic capacity of serum albumin for survival outcome in patients with head and neck ACCs. In particular, it could potentially contribute to identifying patients with a high risk for local or distant metastases, which is a major problem for patients with ACCs [2,3,4]. As blood tests are regularly performed prior to cancer therapy and serum albumin values are usually included in such routine blood analyses, albumin could be easily obtained and would be widely available. Based on the noted study limitations, a validation of our results should be performed in an external cohort.

## Figures and Tables

**Table 1:** Patient and tumor characteristics including treatment modalities.

Age		Years
Mean		57.2
Standard deviation		14.2
**Gender**	*n*	%
Male	16	43.3
Female	21	56.7
**T classification**	*n*	%
T1	4	10.8
T2	11	29.7
T3	8	21.6
T4	14	37.8
**N classification**	*n*	%
N0	31	83.8
N1	6	16.2
N2	0	0
N3	0	0
**M classification**	*n*	%
M0	36	97.3
M1	1	2.7
**Primary therapy**	*n*	%
Surgery alone	13	35.1
Surgery with postoperative radiotherapy	14	37.9
Surgery with postoperative chemoradiotherapy	1	2.7
Radiotherapy	6	16.2
Chemoradiotherapy	2	5.4
Palliative chemotherapy	1	2.7
**Localization**	*n*	%
Minor salivary glands	25	67.6
Sublingual gland	1	2.7
Submandibular gland	6	16.2
Parotid gland	5	13.5

## Data Availability

The data that support the findings of this study are available on request from the corresponding authors (L.K.-W. and F.F.B.).
